# Supplementary figures and images for: The Phenotype and Secretory Activity of Adipose-Derived Mesenchymal Stem Cells (ASCs) of Patients with Rheumatic Diseases
Source: Cells. 2019 Dec 17;8(12):1659. doi: 10.3390/cells8121659 (PMC6952982; doi:10.3390/cells8121659)

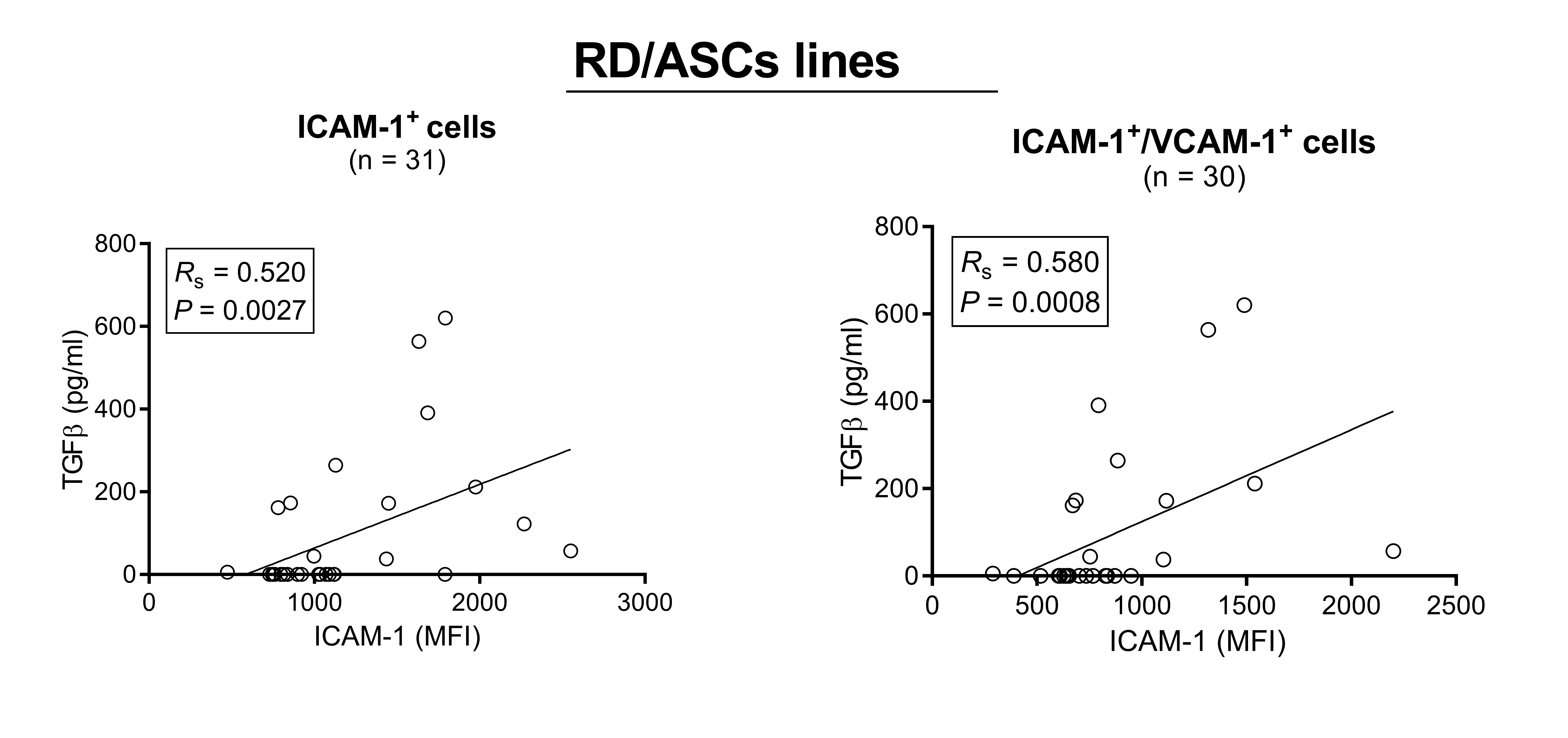

Supplement: Supplementary file 1 [file cells-08-01659-s001.zip › supplemantaryfile/FigureS2.tif]
